# Comparing the Audiological Success of Bone Cement to Standard Ossiculoplasty Techniques: A Systematic Review and Meta‐analysis

**DOI:** 10.1002/ohn.1356

**Published:** 2025-07-22

**Authors:** Natasha Dowell, Shayma Begum, Christopher Coulson, Hannah Nieto, Jameel Muzaffar

**Affiliations:** ^1^ College of Medical and Dental Sciences University of Birmingham Birmingham UK; ^2^ Department of Ear, Nose and Throat Surgery University Hospitals Birmingham NHS Foundation Trust Birmingham UK; ^3^ Institute of Metabolism and Systems Research University of Birmingham Birmingham UK

**Keywords:** bone cement, glass ionomer cement, hydroxyapatite, ossiculoplasty

## Abstract

**Objective:**

To compare the audiological success and longevity of bone cement to standard ossiculoplasty techniques.

**Data Sources:**

PubMed, Embase, Cochrane Library, Medline, and the Web of Science were systematically searched for studies from the date of inception to November 8, 2024.

**Review Methods:**

Two independent reviewers screened for eligibility. Inclusion criteria were full‐text, English language publications. Human studies of any methodology except case series with fewer than five patients were included. The Preferred Reporting Items for Systematic Reviews and Meta‐analyses reporting guidelines were followed. Data were pooled using a random‐effects model using relative risk with 95% CIs. Prespecified primary outcomes compared the success of bone cement reconstruction at short and longer‐term follow‐up.

**Results:**

A total of 27 studies met the eligibility criteria. Bone cement resulted in better audiological outcomes (postoperative mean air‐bone gap < 20 dB HL) compared to incus interposition, risk ratio (RR): 1.26 (95% CI: 1.10‐1.43). There was no difference in success between bone cement and partial ossicular replacement prosthesis (PORP), RR: 1.15 (95% CI: 0.94‐1.41). The findings of this review suggest that bone cement remains successful at a longer‐term follow‐up.

**Conclusion:**

Bone cement outperforms incus interposition and is comparable to PORP for audiological outcomes. Audiological improvement is sustained into longer‐term follow‐up.

Ossicular chain discontinuity is commonly caused by middle ear diseases, primarily chronic otitis media, causing erosion of the long process of the incus.^1^, ^2^ The vulnerability of the incus long process is attributed to its limited blood supply, making it susceptible to necrosis.^3^ This erosion results in incudostapedial joint discontinuity, causing conductive hearing loss. Typically, a maximal conductive loss of 50 to 60 dB HL is observed.^4^ This hearing loss decreases the quality of life for the patient. Although hearing aids are an option, particularly bone conduction devices, which may bypass a large conductive loss, surgical restoration is typically offered to patients through ossiculoplasty. The procedure was first described in 1957 when Hall and Rytzner used autologous ossicles to reestablish the continuity between the stapes and the tympanic membrane.^5^ Since ossiculoplasty was introduced, various procedures and materials have been advocated. A suitable material for ossiculoplasty must be safe, inexpensive, biocompatible, and functional in restoring hearing.^6^ Incus interposition through homologous or autologous sources has been extensively used; however, the graft is vulnerable to becoming displaced. The limited adaptability of incus interposition has led to increasing popularity of partial ossicular replacement prostheses (PORPs). However, extrusion is a potential complication.^7^

The use of bone cement has emerged as a promising alternative to standard techniques. Bone cement offers simple application, osseointegration, and stability.^8^ Primary studies of bone cement ossiculoplasty have typically considered it as an alternative to incus interposition.^9^ The two main types of bone cement are hydroxyapatite bone cement (HABC) and glass ionomer cement (GIC). The choice of bone cement is typically decided by surgeon preference due to the perceived limited difference in stability or success. With the use of bone cement becoming more popular, an increasing number of studies have sought to compare it to standard ossiculoplasty procedures, namely incus interposition and PORP.

A postoperative mean air‐bone gap (ABG) of ≤20 decibel hearing level (dB HL) is commonly recognized in the literature as the standard for assessing ossiculoplasty outcomes.^10^, ^11^, ^12^ Although there is no definitive guideline establishing this threshold, it has been widely adopted in most research as a general measure of success. Although some research has utilized a 10‐dB threshold to indicate exceptional outcomes, the 20‐dB threshold ensures a broader inclusion of studies.^6^ Consequently, this systematic review and meta‐analysis will also use the 20‐dB threshold as the primary criterion for evaluating ossiculoplasty success.

The aim of this systematic review and meta‐analysis is to compare the audiological success and longevity of bone cement to standard ossiculoplasty techniques at a short‐term (<12 months) and longer‐term (≥12 months) follow‐up. This review comments on primary ossiculoplasty alone, and not in combination with stapes revision surgery. This systematic review will compare the audiological success of bone cement to incus interposition and PORP separately.

## Materials and Methods

A systematic review and meta‐analysis based on the Preferred Reporting Items for Systematic Review and Meta‐analyses guidelines was performed.^13^ The review was prospectively registered through the Internal Prospective Register of Systematic Reviews (PROSPERO) (Registration number: CRD42023493041).

### Literature Search and Data Collection

A description of the participants, interventions, comparisons, outcomes, timing, and study design is provided in Supplemental Table S1, available online. Electronic searches were performed in the following databases: PubMed, Embase, Cochrane Library, Medline, and the Web of Science from inception to November 8, 2024. The search strategy was designed with the support of a research librarian. The PubMed search terms included: (Middle ear reconstruction/exp OR auditory ossicle/exp OR ossicular: ti.ab OR “ossic*”: ti.ab OR stapes: ti.ab OR incus: ti.ab OR malleus: ti.ab OR ossiculoplasty: ti.ab OR middle ear: ti.ab) AND (Bone cement: ti.ab OR glass ionomer: ti.ab OR Hydroxyapatite: ti.ab OR Ionomer: ti.ab OR composite: ti.ab OR bone paste: ti.ab OR biocement: ti.ab) AND (Air bone gap [af] OR ABG [af]). Search terms from the additional databases are described in Supplemental Table S2, available online. Two independent reviewers (N.D., S.B.) screened articles for eligibility for inclusion. Disparities between the two reviewers were resolved by a third independent reviewer (J.M./H.N.).

### Eligibility Criteria

The eligibility criteria were as follows:

1. Studies not published in the English language were excluded.
2. Studies had to report the postoperative mean ABG following ossiculoplasty.
3. Only comparative studies with incudostapedial bone cement ossiculoplasty and either incudostapedial interposition, PORP, or both.
4. Studies involving revision cases were excluded.
5. Animal studies were excluded.
6. Systematic reviews, meta‐analyses, literature reviews, and case reports were excluded. Case series were included if they had a sample size of at least five patients.
7. No restrictions were placed on the year of publication.
8. Conference abstracts and studies without full‐text availability were excluded.
9. A minimum postoperative follow‐up period of 3 months was required for suitable comparison.

### Data Extraction

Using a predefined Excel table, two independent reviewers extracted data. Any conflicts were resolved by discussion with a third reviewer (J.M./H.N.).

### Outcome Measures

The outcome measure was audiological success, which was defined as ABG closure to within 20 dB HL. The pure tone scores used to calculate the ABG, and hearing gain should be at the following frequencies: 0.5, 1, 2, and 3 kilohertz (kHz). The guidelines recommend that if the 3‐kHz frequency is missing, an average between 2 and 4 kHz is valid.^14^

### Study Quality

The Downs and Black checklist was used to assess the bias of the included studies.^15^ Any study that scored “poor” overall was excluded from the meta‐analysis.

### Statistical Analysis

Due to methodological heterogeneity, a random‐effects model was used for meta‐analysis, with risk ratio (RR) as the effect measure. Heterogeneity was quantified using *I*_2_ statistics and visualized through forest plots. To evaluate potential publication bias, funnel plots were used with the Egger test of asymmetry.

Statistical analysis was performed in R, Version 4.4.0 (R Project for Statistical Computing).^16^ Meta‐analysis was performed using the “Meta” and “Metafor” packages.^17^, ^18^

## Results

### Study Characteristics

**Figure 1:**
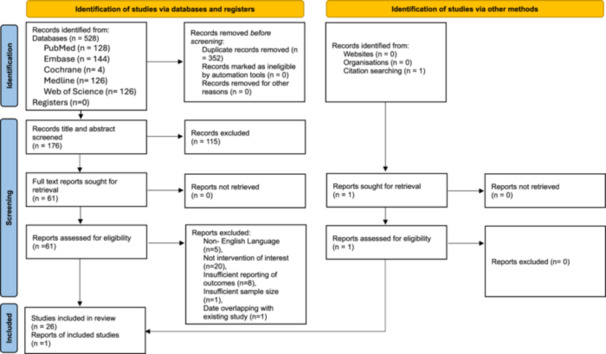
Preferred Reporting Items for Systematic Reviews and Meta‐analyses (PRISMA) flowchart for study selection (1). PRISMA flowchart demonstrating the article screening process based on the eligibility criteria.

Initial searches yielded 517 studies (Figure 1). After removing duplicates, 170 articles were screened, leaving 60 for full‐text review. Of the 27 studies included, 16 (59%) were historical cohort studies and 11 (41%) were case series. The publication years of the included studies covered a 20‐year period between 2004 and 2024. Overall, 1491 patients were included, of whom there were 732 females (53%) and 657 males (47%). Studies included 878 patients who underwent ossiculoplasty with bone cement, 114 received PORP, and 349 received incus interposition. Patient and study characteristics are provided in Tables 1 and 2.

**Table 1:** Study Characteristics

Authors, year	Title	Study design	Tympanic membrane graft material	Surgeon experience	Approach	Type of bone cement
Gülşen and Çikrikci, 2024	Exclusive endoscopic management of incus long process major defects: conventional incus interposition versus malleostapediopexy	Historical cohort	Tragal cartilage and chondroperichondrium	Not discussed	Endoscopic transcanal approach	GIC
Katar et al, 2024	Long term results of glass ionomer ossiculoplasty	Historical case series	Conchal or tragal cartilage grafts	“Experienced surgical team”	Endaural approach	GIC
Moneir et al, 2023	Endoscopic transcanal management of incus long process defects: rebridging with bone cement versus incus interposition	Historical cohort study	Temporalis fascia graft	Not discussed	Endoscopic transcanal approach	GIC
Mohan et al, 2021	Use of glass ionomer cement for incudostapedial rebridging ossiculoplasty	Prospective observational case series	Conchal cartilage or temporalis fascia	Not discussed	Postauricular approach	GIC
Juvekar and Sarkar, 2021	Ossicular reconstruction of incudo‐stapedial joint by glass ionomer—a study of 24 cases	Prospective observational case series	Temporalis fascia graft	Not discussed	Postauricular approach	GIC
Mantsopoulos et al, 2021	Hydroxyapatite bone cement in the reconstruction of defects of the long process of the incus: personal experience and literature review	Historical case series	“Auricular cartilage”	Not discussed	Postauricular approach	HABC
Fatehy and Alzamil, 2020	Bone cement for ossicular chain defects	Prospective case series	Temporalis fascia	Not discussed	Postauricular approach	GIC
Kalcioglu et al, 2020	Are long‐term auditory results following ossiculoplasty with bone cement as successful as early middle period results?	Historical case series	Cartilage or temporal muscle fascia	The same surgeon operated on all cases	Not specified	GIC
Demir et al, 2019	Long‐term outcomes of ossiculoplasty using bone cement	Historical case series	Temporalis muscle fascia or auricular cartilage	Not discussed	Postauricular approach	GIC
Guler and Kum, 2019	Management of incus defects in children: comparison of incus transposition versus glass ionomer cement	Historical cohort study	Temporalis muscle fascia	Senior Surgeon	Postauricular approach	GIC
Yogeesha et al, 2017	Glass ionomer cement: an attractive alternative for the reconstruction of incudo‐stapedial joint discontinuity	Prospective observational case series	Not stated	Not discussed	Postauricular approach	GIC
Atan et al, 2016	Results of ossicular chain reconstruction with glass ionomer cement in paediatric patients	Historical case series	Temporalis muscle fascia or cartilage graft	Not discussed	Postauricular approach	GIC
Edizer et al, 2016	Malleus to stapes bone cement rebridging ossiculoplasty: why don't we perform frequently?	Historical cohort study	Temporalis muscle fascia or tragal cartilage	Not discussed	Not specified	GIC
Gérard et al, 2015	Ossiculoplasty with hydroxyapatite bone cement: our reconstruction philosophy	Historical cohort study	Tragal cartilage with or without perichondrium, fascia temporalis, or Tutopatch	Senior surgeon	Postauricular or endaural approach	HABC
Galy‐bernadoy et al, 2014	Comparison of early hearing outcomes of type 2 ossiculoplasty using hydroxyapatite bone cement versus other materials	Retrospective and prospective cohort	Not stated	Not stated	Postauricular approach	HABC
Baylancicek et al, 2014	Ossicular reconstruction for incus long‐process defects: bone cement or partial ossicular replacement prosthesis	Historical cohort	Tympanic membrane “grafting” not defined	Performed by authors	Endaural or endomeatal approach	GIC
Kalcioglu et al, 2013	The use of bone cement for ossicular chain defects	Historical cohort	Temporalis fascia graft	Not stated	Postauricular approach	GIC
Celenk et al, 2013	Management of incus long process defects: incus interposition versus incudo‐stapedial rebridging with bone cement	Historical cohort study	Temporalis muscle fascia or auricular cartilage	Not stated	Postauricular incision	GIC
Yazici et al, 2013	Comparison of incus interpositioning technique versus glass ionomer cement application in type two tympanoplasty	Historical cohort study	Not stated	Not stated	Postauricular or trans meatal incision	GIC
Somers et al, 2012	Ossicular reconstruction: hydroxyapatite bone cement versus incus remodelling: how to manage incudo‐stapedial discontinuity	Historical cohort study	Not stated	“Performed by the first and last authors”	Postauricular	HABC
Demir et al, 2012	Is it the middle ear disease or the reconstruction material that determines the functional outcome in ossicular chain reconstruction?	Historical cohort study	Not stated	2 senior ear surgeons	Not specified	GIC
Dere et al, 2011	Comparison of glass ionomer cement and incus interposition in reconstruction of incus long process defects	Historical cohort	Temporal fascia or cartilage	Performed by one surgeon	Postauricular incision	GIC
Celik et al, 2009	The impact of fixated glass ionomer cement and springy cortical bone incudo‐stapedial joint reconstruction on hearing results	Historical cohort study	Temporal muscle fascia	Experienced senior authors	Postauricular approach	GIC
Baglam et al, 2009	Incudo‐stapedial rebridging ossiculoplasty with bone cement	Historical case series	Not stated: “tympanic membrane grafting”	Not stated	Postauricular, endaural, trans meatal	GIC
Elsheikh et al, 2006	Physiologic reestablishment of ossicular continuity during excision of retraction pockets: use of hydroxyapatite bone cement for rebridging the incus	Historical cohort study	Tragal cartilage	By the authors	Postauricular approach	HABC
Hafiz, 2005	A more reliable method for incudo‐stapedial rebridging ossiculoplasty: bone cement and wire	Historical cohort	Tragal cartilage	Not stated	Endaural or postauricular	GIC
Babu and Seidman, 2004	Ossicular reconstruction using bone cement	Historical case series	Tragal cartilage	Not stated	Postauricular or trans meatal approach	HABC

Abbreviations: GIC, glass ionomer cement; HABC, hydroxyapatite bone cement.

**Table 2:** Patient Characteristics

Authors	Year	Study population: exclusively pediatric or combined (range: y)	Patient selection method	Indication	Mean age ± SD	Number of patients	Sex	
							Males (n)	Females (n)
Gülşen and Çikrikci	2024	‐	Consecutive (2017‐2022), nonrandom	Chronic otitis media	GIC: 31.2 ± 9.1 Incus interposition: 32.5 ± 11.4	71	33	38
Katar et al.	2024	‐	Consecutive (2015‐2019), nonrandom	Chronic otitis media	‐	40	15	25
Moneir et al.	2023	Combination: range (12‐58)	Consecutive (2016‐2021), nonrandom	Chronic suppurative otitis media	GIC: 32.21 ± 12.26 Incus interposition: 31.78 ± 12.54	83	36	47
Mohan et al.	2021	Combination: range (13‐53)	Non specified, nonrandom	Chronic otitis media	28.04 ± 10.39	25	19	6
Juvekar and Sarkar	2021	Combination: range (15‐55)	Consecutive: 2016‐2018, nonrandom	Chronic otitis media	35.71	24	‐	‐
Mantsopoulos et al.	2021	Combination: range (6.1‐78.8)	Consecutive (2005‐2019), nonrandom	Chronic otitis media, adhesive otitis media, retraction pocket, or limited cholesteatoma	38.10	48	19	29
Fatehy and Alzamil	2020	Combination: range (10‐64)	Consecutive (2015‐2018), nonrandom	Chronic middle ear disease	46.00	30	23	7
Kalcioglu et al.	2020	‐	Consecutive (2004‐2013), nonrandom	Incudostapedial discontinuity, exact indication was not stated	‐	48	9	5
Demir et al.	2019	Combination: range (9‐54)	Consecutive (2006‐2010) nonrandom	Chronic otitis media	34.10 ± 11.80	40	16	24
Guler and Kum	2019	Pediatric only: range (10‐16)	Consecutive (2010‐2017), nonrandom	Chronic otitis media	Total: 12.97 ± 1.59 GIC: 13.27 ± 1.40 Incus interposition: 12.72 ± 1.72	40	21	19
Yogeesha et al.	2017	Combination: range (15‐55)	Consecutive (2012‐2014), nonrandom	Chronic suppurative otitis media	31.63 ± 10.96	27	15	12
Atan et al.	2016	Pediatric only: range (10‐16)	Consecutive (2009‐2015), nonrandom	Chronic otitis media	13.26 ± 1.90	15	8	7
Edizer et al.	2016	Combination: range (11‐67)	Consecutive (2008‐2014), nonrandom	Chronic otitis media, cholesteatoma	35.17 ± 12.76	92	33	59
Gérard et al.	2016	‐	Consecutive: (2007‐2013), nonrandom	Chronic otitis media, cholesteatoma, otosclerosis, trauma, congenital ossicular malformation	‐	127	‐	‐
Galy‐bernadoy et al.	2014	Combination: range (11‐78)	Consecutive: (2007‐2012), nonrandom	Chronic otitis media only	45.80	70	36	34
Baylancicek et al.	2014	Combination: range (15‐37)	Consecutive: (2008‐2012), nonrandom	Incudostapedial discontinuity	GIC: 33.30 ± 9.80 PORP: 27.30 ± 15.70	44	31	36
Kalcioglu et al.	2013	Combination: range (10‐65)	Consecutive (2000‐2011), nonrandom	Chronic otitis media	32.00	67	‐	‐
Celenk et al.	2013	Combination: range (8‐67)	No mention of consecutive, but assumed nonrandom	Chronic otitis media, adhesive otitis media	GIC: 29.10 ± 14.89 Incus interposition: 29.43 ± 12.50	99	38	61
Yazici et al.	2013	‐	Consecutive (2005‐2008), nonrandom	Chronic otitis media	GIC: 36.0 ± 13.5 Incus interposition: 30.2 ± 14.9	107	43	64
Somers et al.	2012	Combination: range (6‐68)	Consecutive, nonrandom	Incudostapedial discontinuity	HABC: 35.8 Incus interposition: 33.60	22 patients, 24 ears	9	13
Demir et al.	2012	Combination: range (11‐70)	Consecutive (2007‐2010), nonrandom	Chronic otitis media, cholesteatoma	34.80	23	64	46
Dere et al.	2011	Combination: range (14‐66)	Consecutive (2003‐2009), random selection	Incus long process defect	GIC: 31.00 Incus interposition: 31.00	46	17	29
Celik et al.	2009	Combination: range (12‐57)	Consecutive: 1996‐2008), nonrandom	Chronic otitis media and long process defect	36.00 ± 11.00	66	27	39
Baglam et al.	2009	Combination: range (14‐62)	Consecutive (2000‐2007), nonrandom	Chronic otitis media, retraction pockets	30.96	136	64	72
Elsheikh et al.	2006	‐	Consecutive (2002‐2004), nonrandom	Chronic otitis media, retraction pockets	HABC: 38.00 ± 7.80 PORP: 41.00 ± 8.20	82 ears of 62 patients	61	41
Hafiz	2005	Combination: range (13‐60)	Consecutive (1999‐2003), nonrandom	Incudostapedial joint discontinuity	35.00	21	13	8
Babu and Seidman	2004	Combination: range (11‐70)	Consecutive (1999‐2004), nonrandom	Chronic suppurative otitis media	35.78	18	7	11

Abbreviations: GIC, glass ionomer cement; HABC, hydroxyapatite bone cement; PORP, partial ossicular replacement prosthesis.

### Reporting Quality

The overall quality assessment scores for the Downs and Black checklist are provided in Supplemental Table S3, available online. The scores ranged from 15 to 21 out of a total score of 28. Twenty‐six studies were categorized as fair quality, and one paper was categorized as good quality. No studies were excluded after quality scoring as no studies were categorized as poor quality overall.

### Audiological Outcomes

Audiological data were extracted and provided in Table 3 and Supplemental Table S4, available online. The studies containing a non‐bone cement group included various techniques including incus interposition,^6^, ^9^, ^19^, ^20^, ^21^, ^22^, ^23^, ^24^, ^25^ PORP,^21^, ^26^, ^27^, ^28^ and bone cement with wire.^29^, ^30^ Of the bone cement groups, 21 of the 27 included studies used GIC and 6 studies used HABC.

**Table 3:** Audiological Data

				ABG closure to within 20 dB HL			Mean hearing gain, dB HL ± SD		
Authors, year	Treatment group	Control group (s)	Mean follow‐up duration, mo	Treatment %	Control %	Risk difference %	Treatment	Control	Mean difference
Gülşen and Çikrikci, 2024	GIC (34)	Incus interposition (37)	24.2 ± 5.1	88.2	70.3	17.9	18.3 ± 5.1	14.7 ± 4.2	3.6
Katar et al, 2024	GIC (40)	‐	3 44.5	60 ‐	‐	‐	17.34 17.8	‐	‐
Moneir et al, 2023	GIC (38)	Incus interposition (45)	6	81.60	71.10	10.50	21.39 ± 2.15	19.71 ± 6.12	1.68
Mohan et al, 2021	GIC (25)	‐	4	76	‐	‐	10.8	‐	‐
Juvekar and Sarkar, 2021	GIC (24)	‐	(range 3‐12)	91.67	‐	‐	12.5	‐	‐
Mantsopoulos et al, 2021	HABC (48)	‐	Short term: 6‐12 Long term: 12	Short term: 83	‐	‐	Short term: 9.55 Long term: 10.26	‐	‐
Fatehy and Alzamil, 2020	GIC (30)	‐	12	52	‐	‐	22.2	‐	‐
Kalcioglu et al, 2020	GIC (14)	‐	24 and 102	‐	‐	‐	24 months: 15.625 102 months: 4.68	‐	‐
Demir et al, 2019	GIC (40)	‐	62.4 ± 5.1	82.50	‐	‐	8	‐	‐
Guler and Kum, 2019	GIC (18)	Incus interposition (22)	6	94.40	63.60	30.80	18.83 ± 9.43	10.31 ± 9.44	8.52
Yogeesha et al, 2017	GIC (27)	‐	3	66.67	‐	‐	13.25	‐	‐
Atan et al, 2016	GIC: (15)	‐	12	40	‐	‐	20.33 ± 6.36	‐	‐
Edizer et al, 2016	IS‐BCR (42)	Incus interposition (18)	12	IS: 54.7	55.5	−0.8	12.9	17.4	−4.5
Gérard et al, 2015	HABC (40)	PORP (57)	26 ± 14	HABC: 95	PORP: 82.5	‐	HABC: 16.2 ± 12.9	PORP: 13.8 (16.6)	PORP: 2.4
Galy‐bernadoy et al, 2014	HABC (11)	Cartilage (14)	3	90.91	57.14	33.77	15	10.63	4.37
		Incus interposition (11)			44.45	45.46		12.25	2.75
		Titanium PORP (14)			78.57	12.34		13.72	1.28
Baylancicek et al, 2014	GIC (21)	PORP (23)	12	90.4	86.9	3.5	16.85 ± 10.59	20.6 ± 9.38	−3.75
Kalcioglu et al, 2013	GIC (29)	Incus interposition (25)	35 (range: 6‐86)	76	64	12	17.5	15.8	1.7
		Autograft (13)			46	30		22.3	−4.8
Celenk et al, 2013	GIC (50)	Incus interposition (49)	GIC: 25.8 ± 1.01 Incus interposition: 26.13 ± 1.1	78	63.20	14.80	19.36 ± 9.08	15.2 ± 9.01	4.2
Yazici et al, 2013	GIC (28)	Incus interposition (79)	9.8 (6‐38)	78.60	43	35.60	17.0 ± 11.96	11.0 ± 13.02	6
Somers et al, 2012	HABC (10)	Incus interposition (14)	Intervals at 3, 6, and 12	3 months: 90 6 months: 80 12 months: 80	3 months: 71.4 6 months: 64.3 12 months: 57.1	3 months: 18.6 6 months: 15.7 12 months: 22.9	3 months: 19.9 6 months: 20 12 months: 18.4	3 months: 16.7 6 months: 11.3 12 months: 4.4	3 months: 3.2 6 months: 8.7 12 months: 14
Demir et al, 2012	GIC (23)	Incus interposition (8)	23.6 (12‐56)	‐	‐	‐	8.05	11.0	−2.95
		TORP/PORP (2)						7.5	0.55
Dere et al, 2011	GIC (23)	Incus interposition (23)	12	‐	‐	‐	6.3	8.5	(−2.2)
Celik et al, 2009	GIC (31)	Incus interposition (35)	Overall: 22.8 ± 9.1 GIC: 17.5 ± 9.3 Incus interposition: 27.5 ± 5.8	94	86	8	19.1 ± 7	17.4 ± 7.1	1.7
Baglam, et al, 2009	GIC (136)	‐	12	82	‐	‐	19.29 ± 5.578	‐	‐
Elsheikh et al, 2006	HABC (48)	PORP (20)	21, audiology at 13 months	92	55	27	18.9 ± 11	22.4 ± 9.2	−3.4
Hafiz, 2005	GIC (15)	Bone cement and wire (6)	12	76	‐	‐	27.3	32.3	−5
Babu and Seidman, 2004	HABC (18)	‐	12 to 36	94.44	‐	‐	23	‐	‐

Abbreviations: ABG, air‐bone gap; GIC, glass ionomer cement; HABC, hydroxyapatite bone cement; IS, incudostapedial; IS‐BCR, incudostapedial bone cement re‐bridging; PORP, partial ossicular replacement prosthesis; TORP, total ossicular replacement prosthesis.

**Figure 2:**
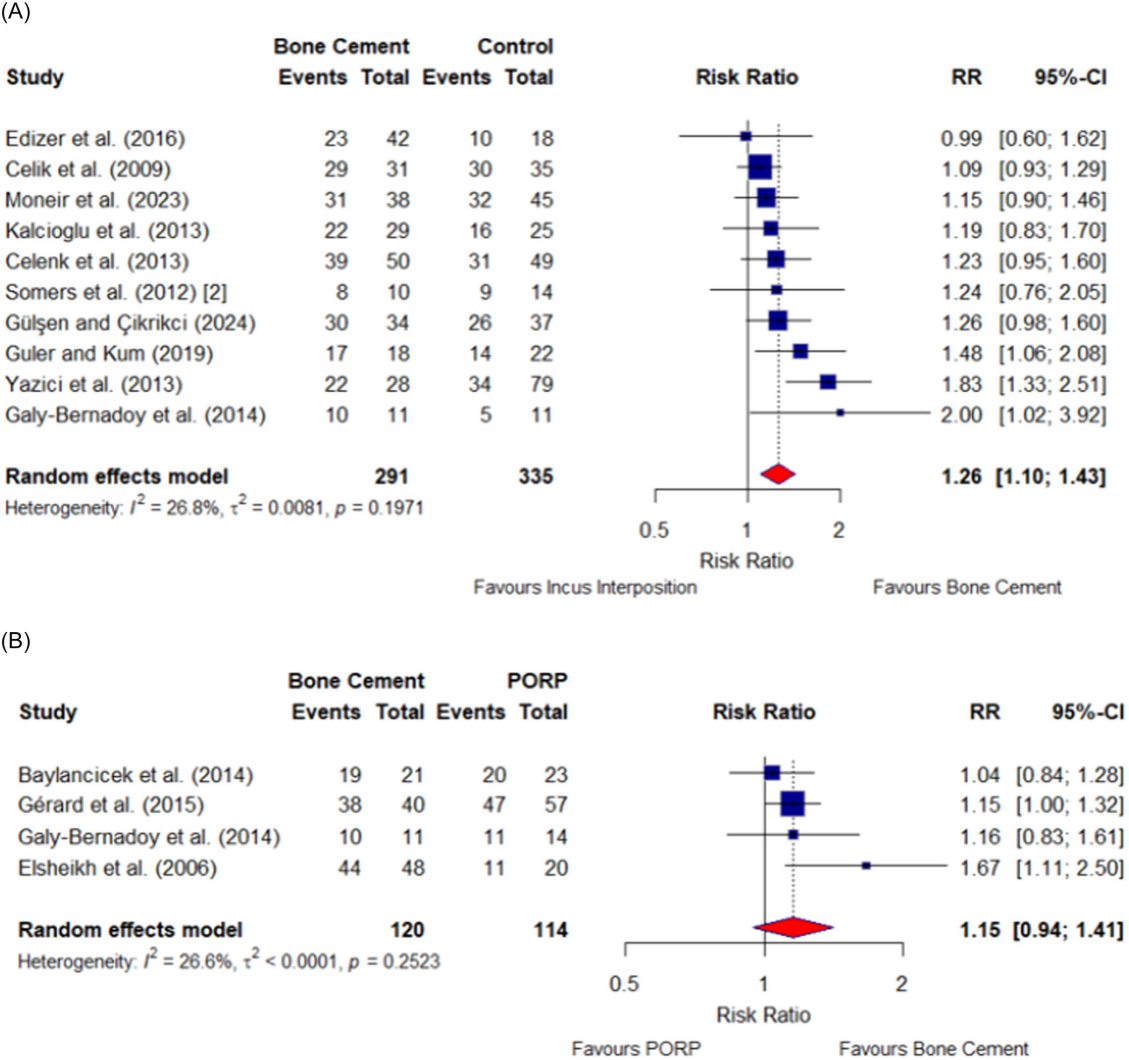
Meta‐analysis comparing ossiculoplasty success: bone cement versus incus interposition (A) and partial ossicular replacement prosthesis (PORP) (B). Control is incus interposition; squares show study weight; bars show 95% CIs; and diamonds show pooled risk ratio. RR, risk ratio.

A meta‐analysis was conducted to compare the audiological success between the bone cement and non‐bone cement groups, namely incus interposition or PORP. In total, 10 studies compared between bone cement and incus interposition and provided a postoperative mean ABG ≤ 20 dB HL. Regarding the primary outcome, there was a significant difference in the RR showing bone cement to have greater audiological success to incus interposition (RR, 1.26 [95% CI, 1.10‐1.43]; *I* ^2^ = 26.8%) (Figure 2A).

Four studies reported the proportion of patients achieving a postoperative ABG ≤ 20 dB HL when comparing between a bone cement and PORP. There was no significant difference in the RR comparing the audiological success (proportion of patients achieving a postoperative ABG ≤ 20 dB HL) of bone cement to PORP (RR, 1.15 [95% CI, 0.94‐1.41]; *I* ^2^ = 26.6%) (Figure 2B).

### Comparing Follow‐Up Duration Between Bone Cement and Controls

**Figure 3:**
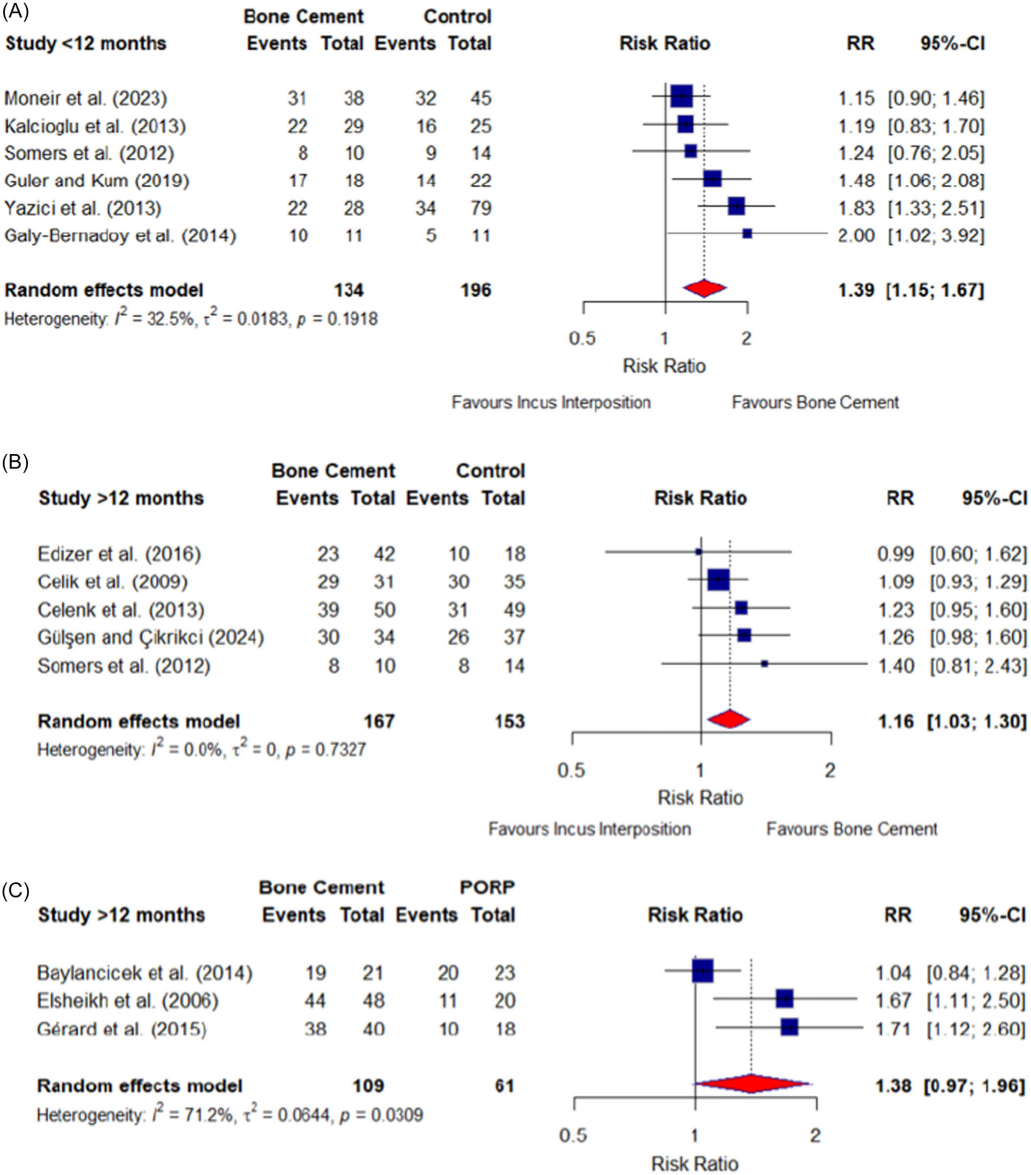
Meta‐analysis of ossiculoplasty success: bone cement versus controls. (A) Interposition (<12 months), (B) interposition (>12 months), and (C) partial ossicular replacement prosthesis (PORP) (>12 months). Control indicates incus interposition. Error bars indicate 95% CIs of risk ratio (RR).

Seven studies compared audiological success between bone cement and standard ossiculoplasty procedures over a short‐term follow‐up period (<12 months). Six studies compared bone cement to incus interposition. There was a significant difference in the RR showing bone cement to have greater audiological success to incus interposition up to a 1‐year postoperative period (RR, 1.39 [95% CI, 1.15‐1.67]; *I* ^2^ = 32.5%) (Figure 3A).

Five studies compared audiological success between bone cement and incus interposition over a follow‐up period greater than 12 months.^6^, ^12^, ^19^, ^20^, ^25^ The forest plot reported a significant difference in the RR showing bone cement to have greater audiological success to the incus interposition at a follow‐up period greater than or equal to 12 months (RR, 1.16 [95% CI, 1.03‐1.30]; *I* ^2^ = 0%) (Figure 3B).

Only one study compared bone cement to PORP at a follow‐up of less than 12 months, providing insufficient data to perform a meta‐analysis at this time point.^21^ In contrast, three studies compared bone cement to PORP beyond 12 months.^26^, ^27^, ^28^ The pooled analysis demonstrated no statistically significant difference in the RR between bone cement and PORP beyond 12 months (RR, 1.38 [95% CI, 0.97‐1.96]; *I* ^2^ = 71.2%) (Figure 3C).

### Publication Bias

Visual inspection of the funnel plot showed no signs of asymmetry (Supplemental Figure S1, available online). There was no evidence of small study effect of publication bias (Egger test, *t* = 1.85, *P* = .0891).

## Discussion

This systematic review and meta‐analysis compared the hearing outcomes between bone cement (HABC, GIC) to standard ossiculoplasty techniques (PORPs and incus interposition). Bone cement has emerged as an alternative to these procedures, by offering a simple procedure with great biocompatibility, audiological success, and low risk of extrusion.

Both HABC and GIC are widely used in ossiculoplasty with numerous cohort studies discussing their stability, as included in this review. There is little evidence to suggest any differences in outcomes between the two types of bone cement. However, further literature should perform a direct comparison to determine if either bone cement is superior to the other in terms of stability and overall effectiveness. Although recent literature has not compared between bone cement types, it has focused on assessing the effectiveness of bone cement compared to incus interposition and PORP, as well as its stability across varying follow‐up periods.

This systematic review and meta‐analysis is the first study to compare bone cement to incus interposition and PORP separately. This study found that bone cement outperforms incus interposition but shows no advantage over PORPs. It is worth noting that 10 studies compared bone cement to incus interposition, but only 4 studies compared bone cement to PORP, highlighting that further research is needed in this area.

A meta‐analysis was not performed for the bone cement and wire group due to their being limited study numbers comparing this group to a standard technique. Two earlier studies Hafiz^29^ and Babu and Seidman^30^ discussed this technique as a case series and were included in the review to provide context on the earlier use of bone cement and to demonstrate its audiological success and clinical application.

Two previous systematic reviews were conducted in this area of research; however, both pooled primary ossiculoplasty and stapes revision cases, whereas our review excluded stapes surgery and revision cases of other ossiculoplasty. The first systematic review to compare bone cement to standard ossiculoplasty techniques was performed by Wegner et al.^31^ Their review included a combination of case series and cohort studies and reported a narrative synthesis that there was no evidence to suggest that bone cement was any more successful than conventional materials. A more recent systematic review was published by Reis et al,^32^ which concluded that bone cement had greater audiological success than standard ossiculoplasty procedures (odds ratio [OR] = 2.03, *P* = .001). This review combined the control groups across the papers, so that incus interposition and PORP were compared together to bone cement.^32^ This meant that no individual conclusion was drawn between bone cement and incus interposition and bone cement and PORP.

The secondary aim of this study was to compare the longevity of bone cement to its comparators. This review compared a short‐term follow‐up duration (<12 months) to a longer‐term follow‐up duration (≥12 months). This study identified that bone cement remained successful between the two periods and therefore holds longevity. The meta‐analysis also demonstrated that bone cement holds greater audiological outcomes compared to incus interposition at a short‐ and longer‐term follow‐up period, whereas there is no difference between bone cement and PORP beyond 12 months.

The decision to categorize follow‐up periods as less than 12 months or 12 months or greater was based on the follow‐up durations reported in the included studies. Although longer‐term analysis (5‐10 years) would have been preferable, most available data came from case series rather than cohort studies. Although follow‐up periods beyond 12 months are not typically considered long‐term for surgical outcomes, this review included case series to assess the long‐term stability of bone cement. Although earlier studies raised concerns about the long‐term stability of GIC, with a mean survival of 28 months and cases of extrusion, our review suggests longer‐term stability.^33^ Among the 11 case series included, most demonstrated sustained success of bone cement at longer follow‐up periods.^34^, ^35^, ^36^, ^37^, ^38^, ^39^, ^40^, ^41^ Notably, Katar et al.^42^ reported a mean follow‐up of 44.5 months, and Demir et al.^43^ reported 62.4 months, both showing sustained outcomes without widespread extrusion.

### Indication for Ossiculoplasty and Surgical Approach

All 27 included studies had chronic otitis media as an indication, with 15 studies focusing exclusively on this. The remaining studies included a range of indications, including incudostapedial discontinuity, cholesteatoma, trauma, and congenital ossicular malformation. Most studies did not isolate cholesteatoma or congenital malformations as distinct groups, with only four studies including cholesteatoma patients; therefore, it was not possible to subgroup the indications. Moreover, chronic otitis media and cholesteatoma are associated with eustachian tube dysfunction, whereas congenital ossicular malformation is not, which could impact the comparability between the studies.^44^ These differences are a limitation of this review, and future research should aim to isolate indications to assess their impact on ossiculoplasty outcomes.

In total, 16 studies reported the graft used in tympanic membrane reconstruction, which included temporalis fascia or tragal cartilage. There is limited evidence to suggest either material is superior to one another in terms of audiological outcomes, and therefore, the reconstruction material is unlikely to influence the results of the studies.^45^

Regarding surgical approach, 20 studies used a postauricular incision, whereas the remaining studies used either an endoscopic transcanal or endaural approach. Although differences in outcomes may arise from various surgical approaches, most studies performed a postauricular incision, meaning it is unlikely that this factor influenced the outcomes between papers. It would have been appropriate to compare between different approaches; however, there was a limited number of studies that did not use a postauricular incision, and therefore, there would not be a sufficient sample size for a meta‐analysis to compare surgical approaches.

### Strengths and Limitations

The study's strengths lie in its rigorous methodology and robust evidence synthesis. The main limitation is the historical nature and methodological design of the included studies. The articles varied in their indication for treatment and extent of incudostapedial discontinuity. Historical data introduce the potential for selective reporting of information, which affects the reliability and findings drawn from this review.

A limitation of this review is the lack of consistent reporting on the number and experience of surgeons performing the ossiculoplasty procedures. Only 10 studies provided surgeon details, and 13 studies offered no information on whether the same surgeon performed the procedures or their experience level. This lack of standardization may influence the results of our analysis.

### Implications for Practice

The nuanced understanding of the audiological success and longevity of bone cement will support clinical decision‐making. Anatomical factors and surgical preference typically guide the selection of the most appropriate technique for ossiculoplasty. Information derived from evidence synthesis can help to guide patient selection and counseling. Although our work includes hearing outcomes at more than 12 months, it is important that future studies explore longer‐term outcomes.

## Conclusions

Bone cement is an advantageous material due to its biocompatibility, simple application, and its ability to provide successful hearing outcomes. This review shows that bone cement has greater audiological success compared to incus interposition but is of no difference to PORP's. Studies with a longer duration of follow‐up are essential to guide long‐term counseling.

## Author Contributions

**Natasha Dowell**, Contribution to the design of the work, first reviewer, literature searches, data extraction, statistics and writing of the paper. **Shayma Begum**, Literature searches, second reviewer, data extraction. **Christopher Coulson**, Project management + accountability, revising and editing document. **Hannah Nieto**, Helped design SR, supervised project, critically appraised data and writing, helped draft, edited writing. **Jameel Muzaffar**, Suggestion of study question, refinement of protocol, revision of text, responsibility for content, supervised project.

## Disclosures

### Competing interests

There are no conflicts of interest to declare.

### Funding source

N.D.: Topham Bursary, University of Birmingham.

## Supporting information

Supporting Information.
