# Supplementary material for: Comparing the Audiological Success of Bone Cement to Standard Ossiculoplasty Techniques: A Systematic Review and Meta‐analysis
Source: Otolaryngol Head Neck Surg. 2025 Jul 22;173(5):1054–68. doi: 10.1002/ohn.1356 (PMC12574626; doi:10.1002/ohn.1356)
Supplement: Supplementary file 1 — Supporting Information. [file OHN-173-1054-s001.docx]

**
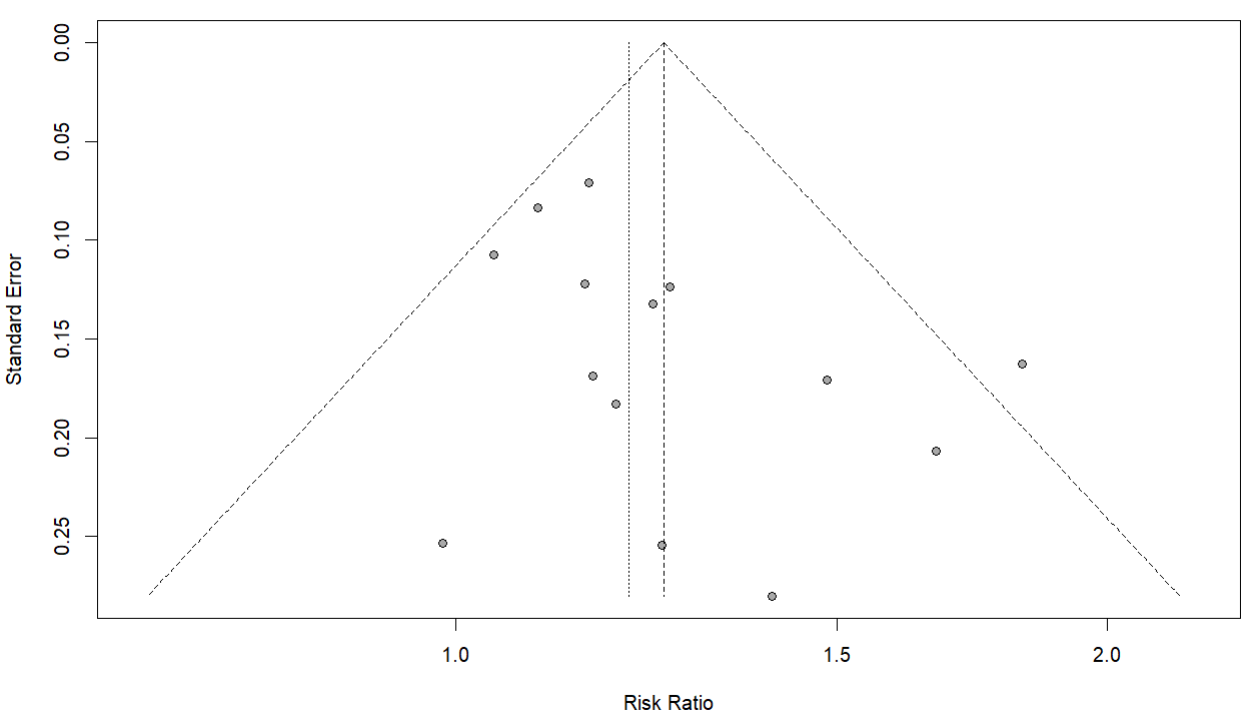
Supplemental Figure 1: Funnel plot of studies**

A funnel plot visualising publication bias of the studies comparing bone cement to standard ossiculoplasty techniques.

**Supplemental Table 1: Description of study criteria**

| PICOTS | Description |
| --- | --- |
| Population | Adults and children of any age with ossicular chain discontinuity requiring ossiculoplasty |
| Interventions | Ossiculoplasty using any type of bone cement |
| Comparisons | Standard ossiculoplasty techniques including but not limited to PORP or incus interposition |
| Outcomes | Post operative mean ABG ≤20 dB HL |
| Timing | Any time point |
| Study Design | A Systematic Review and Meta-Analysis |

| **Supplemental Table 2: Systematic Search Strategy** | | |
| --- | --- | --- |
| **#** | **Database** | **Search Terms** |
| 1 | PubMed | (Middle ear [MeSH Major Topic] OR Ossicular replacement [MeSH major topic] OR ossiculoplasty [title/abstract] OR ossic* [Title/abstract] OR stapes [Title/abstract] OR malleus [title/abstract] OR incus [title/abstract] OR middle ear[title/abstract]) AND (Bone cement [MeSH Major topic] OR Bone cement* [title/abstract] OR glass ionomer [title/abstract] OR hydroxyapatite [title/abstract] OR ionomer [title/abstract] OR composite [title/abstract] OR biocement [title/abstract] OR bone paste [title/abstract]) AND (Air bone gap [title/abstract] OR ABG [title/abstract]) |
| 2 | Embase and Ovid-Medline | (Middle ear reconstruction/exp OR auditory ossicle/exp OR ossicular: ti.ab OR “ossic*”: ti.ab OR stapes: ti.ab OR incus: ti.ab OR malleus: ti.ab OR ossiculoplasty: ti.ab OR middle ear: ti.ab) AND (Bone cement: ti.ab OR glass ionomer: ti.ab OR Hydroxyapatite: ti.ab OR Ionomer: ti.ab OR composite: ti.ab OR bone paste: ti.ab OR biocement: ti.ab) AND (Air bone gap [af] OR ABG [af]) |
| 3 | Cochrane Library | (MeSH descriptor: [Ear Ossicles] explode all trees OR MeSH descriptor: [Ear, Middle] explode all trees OR ossic* OR stapes OR incus OR malleus OR ossiculoplasty OR Middle ear) AND (MeSH descriptor: [Bone cements] explode all trees OR bone cement* OR glass ionomer cement OR hydroxyapatite OR ionomer) AND (Air bone gap OR ABG) |
| 4 | Web of Science | (Ossicular [tiab] OR Ossic* [tiab] OR stapes [tiab] OR incus [tiab] OR malleus [tiab] OR ossiculoplasty [tiab] OR middle ear [tiab]) AND (Bone cement [tiab] OR glass ionomer [tiab] OR hydroxyapatite [tiab] OR ionomer [tiab] OR composite [tiab] PR bone paste [tiab] OR biocement [tiab]) AND (Air bone gap[all] OR ABG [all]) |

| **Supplemental Table 3: Study Quality and Risk of Bias using the Downs and Black Checklist** | | | | | | | | |
| --- | --- | --- | --- | --- | --- | --- | --- | --- |
| **Author** | **Year** | **Reporting** | **External validity** | **Internal validity- bias** | **Internal validity- confounding** | **Power** | **Total Score** | **Quality category** |
| Gülşen and Çikrikci (1) | 2024 | 10 | 2 | 3 | 2 | 0 | 17 | **Fair** |
| Katar et al (2) | 2024 | 10 | 2 | 3 | 3 | 0 | 18 | **Fair** |
| Moneir et al (3) | 2023 | 11 | 3 | 4 | 3 | 0 | 21 | **Good** |
| Mohan et al (4) | 2021 | 10 | 2 | 4 | 2 | 0 | 18 | **Fair** |
| Juvekar and Sarkar (5) | 2021 | 10 | 2 | 4 | 2 | 0 | 18 | **Fair** |
| Mantsopoulos et al (6) | 2021 | 8 | 2 | 4 | 2 | 0 | 16 | **Fair** |
| Fatehy and Alzamil (7) | 2020 | 8 | 2 | 4 | 2 | 0 | 16 | **Fair** |
| Kalcioglu et al (8) | 2020 | 8 | 2 | 3 | 2 | 0 | 15 | **Fair** |
| Demir et al (9) | 2019 | 10 | 2 | 3 | 3 | 0 | 18 | **Fair** |
| Guler and Kum (10) | 2019 | 10 | 2 | 3 | 3 | 0 | 18 | **Fair** |
| Yogeesha et al (11) | 2017 | 10 | 2 | 4 | 2 | 0 | 18 | **Fair** |
| Atan et al (12) | 2016 | 10 | 2 | 3 | 2 | 0 | 17 | **Fair** |
| Edizer et al (13) | 2016 | 9 | 2 | 4 | 3 | 0 | 18 | **Fair** |
| Gérard et al (14) | 2015 | 8 | 2 | 3 | 2 | 0 | 15 | **Fair** |
| Galy- bernadoy et al (15) | 2014 | 10 | 2 | 4 | 2 | 0 | 18 | **Fair** |
| Baylancicek et al (16) | 2014 | 10 | 2 | 4 | 3 | 0 | 19 | **Fair** |
| Kalcioglu, Tan, Fleerakkers (17) | 2013 | 9 | 2 | 3 | 3 | 0 | 17 | **Fair** |
| Celenk et al (18) | 2013 | 10 | 2 | 3 | 3 | 0 | 18 | **Fair** |
| Yazici et al (19) | 2013 | 10 | 2 | 3 | 3 | 0 | 18 | **Fair** |
| Somers et al (20) | 2012 | 10 | 2 | 4 | 3 | 0 | 19 | **Fair** |
| Demir et al (21) | 2012 | 10 | 2 | 4 | 2 | 0 | 18 | **Fair** |
| Dere et al (22) | 2011 | 8 | 2 | 4 | 4 | 0 | 18 | **Fair** |
| Celik et al (23) | 2009 | 10 | 2 | 3 | 3 | 0 | 18 | **Fair** |
| Baglam et al (24) | 2009 | 10 | 2 | 4 | 2 | 0 | 18 | **Fair** |
| Elsheikh, Elsherief and Elsherief (25) | 2006 | 10 | 2 | 4 | 2 | 0 | 18 | **Fair** |
| Hafiz (26) | 2005 | 10 | 2 | 4 | 2 | 0 | 18 | **Fair** |
| Babu and Seidman (27) | 2004 | 9 | 2 | 3 | 2 | 0 | 16 | **Fair** |

| Supplemental Table 4 | | |
| --- | --- | --- |
| Author | Year | Complications |
| Gülşen and Çikrikci (1) | 2024 | No facial nerve related complications  Temporary dysgeusia in 18.95 of incus interposition group and in 23.5% of the GIC group. |
| Katar, et al (2) | 2024 | No complications associated to GIC use. |
| Moneir, et al (3) | 2023 | Taste disturbance:  GIC:(n= 2) Incus interposition (n= 3) |
| Mohan, et al (4) | 2021 | 1 - failure of graft uptake |
| Juvekar and Sarkar (5) | 2021 | No complications experienced |
| Mantsopoulos et al (6) | 2021 | Not discussed |
| Fatehy and Alzamil (7) | 2020 | No serious complications were experienced |
| Kalcioglu et al (8) | 2020 | Not discussed |
| Demir et al (9) | 2019 | No complications were observed. |
| Guler and Kum (10) | 2019 | No complications were associated with the use of GIC. |
| Yogeesha et al (11) | 2017 | No complications were observed |
| Atan et al (12) | 2016 | No major postoperative complications |
| Edizer et al (13) | 2016 | Not discussed |
| Gérard et al (14) | 2015 | No complications related to the cement or extrusion occurred. |
| Galy- bernadoy, et al (15) | 2014 | No major complications reported |
| Baylancicek, et al (16) | 2014 | No complications to do with bone cement or extrusion |
| Kalcioglu, Tan, Fleerakkers (17) | 2013 | Not discussed |
| Celenk et al (18) | 2013 | No serious complications 2 patients required revision surgery: Bone cement separation (n=1) Granulation tissue around ossicles (n=1) |
| Yazici, et al (19) | 2013 | No adverse effects from GIC |
| Somers, et al (20) | 2012 | Not discussed |
| Demir et al (21) | 2012 | Not discussed |
| Dere, et al (22) | 2011 | No complications observed. |
| Celik, et al (23) | 2009 | No major or minor complications observed |
| Baglam, et al (24) | 2009 | No complications observed. |
| Elsheikh, Elsherief and Elsherief (25) | 2006 | 2 - extrusion of prothesis, 4 - recurrent CHL |
| Hafiz (26) | 2005 | 2 failed and required revision |
| Babu and Seidman (27) | 2004 | No complications observed. |

eReferences:

1. Gülşen S, Çikrikci S. Exclusive endoscopic management of incus long process major defects: conventional incus interposition versus malleostapediopexy. *Eur Arch Otorhinolaryngol*. 2024.
2. Katar O, Kılıç S, Bajin MD, Sennaroğlu L. Long term results of glass ionomer ossiculoplasty. *Eur Arch Otorhinolaryngol*. 2024;281(1):171-9.
3. Moneir W, Salem MA, Hemdan A. Endoscopic transcanal management of incus long process defects: rebridging with bone cement versus incus interposition. *Eur Arch Otorhinolaryngol*. 2023;280(2):557-63.
4. Mohan A, Bhagat S, Sahni D, Kaur G. Use of Glass Ionomer Cement for Incudostapedial Rebridging Ossiculoplasty. *Iran J Otorhinolaryngol.* 2021;33(115):65-70.
5. Juvekar M, B S. Ossicular reconstruction of incudo-stapedial joint by glass ionomer—a study of 24 cases. *The Egyptian Journal of Otolaryngology.* 2021;37(1).
6. Mantsopoulos K, Thimsen V, Wohlleben F, Taha L, Sievert M, Müller SK, et al. Hydroxyapatite bone cement in the reconstruction of defects of the long process of the incus: Personal experience and literature review. *Am J Otolaryngol.* 2021;42(4):103002.
7. Fatehy E, Alzamil W. Bone Cement for Ossicular Chain Defects. *The Medical Journal of Cairo University*. 2020;88(9):1753-9.
8. Kalcioglu MT, Yalcin MZ, Kilic O, Tuysuz O, Tan M, Ozdamar OI. Are long-term auditory results following ossiculoplasty with bone cement as successful as early-middle period results? *Am J Otolaryngol.* 2020;41(6):102620.
9. Demir B, Binnetoglu A, Sahin A, Derinsu U, Batman Ç. Long-term outcomes of ossiculoplasty using bone cement*. J Laryngol Otol.* 2019;133(8):658-61.
10. Guler I, Kum RO. Management of Incus Defects in Children: Comparison of Incus Transposition Versus Glass Ionomer Cement*. Ear Nose Throat J*. 2021;100(2):97-102.
11. Yogeesha B, Rohit K, Maradi N. Glass ionomer cement: An attractive alternative for the reconstruction of incudostapedial joint discontinuity. *Indian Journal of Otology.* 2017;23:222.
12. Atan D, Dere H, Yamur AR, Özcan KM. Results of ossicular chain reconstruction with glass ionomer cement in pediatric patients*. Int J Pediatr Otorhinolaryngol.* 2016;85:103-6.
13. Edizer DT, Durna YM, Hamit B, Demirhan H, Yigit O. Malleus to Stapes Bone Cement Rebridging Ossiculoplasty: Why Don't We Perform Frequently? *Ann Otol Rhinol Laryngol.* 2016;125(6):445-51.
14. Gérard JM, De Bie G, Franceschi D, Deggouj N, Gersdorff M. Ossiculoplasty with hydroxyapatite bone cement: our reconstruction philosophy*. Eur Arch Otorhinolaryngol.* 2015;272(7):1629-35.
15. Galy-Bernadoy C, Akkari M, Mathiolon C, Mondain M, Uziel A, Venail F. Comparison of early hearing outcomes of type 2 ossiculoplasty using hydroxyapatite bone cement versus other materials. *Eur Ann Otorhinolaryngol Head Neck Dis.* 2014;131(5):289-92.
16. Baylancicek S, Iseri M, Topdağ D, Ustundag E, Ozturk M, Polat S, et al. Ossicular reconstruction for incus long-process defects: bone cement or partial ossicular replacement prosthesis*. Otolaryngol Head Neck Surg.* 2014;151(3):468-72.
17. Kalcioglu MT, Tan M, Fleerakkers J. The use of bone cement for ossicular chain defects. *Eur Arch Otorhinolaryngol*. 2013;270(11):2849-55.
18. Celenk F, Baglam T, Baysal E, Durucu C, Karatas ZA, Mumbuc S, et al. Management of incus long process defects: incus interposition versus incudostapedial rebridging with bone cement. *J Laryngol Otol.* 2013;127(9):842-7.
19. Yazıcı H, Uzunkulaoğlu H, Emir HK, Kızılkaya Z, Doğan S, Samim E. Comparison of incus interpositioning technique versus glass ionomer cement application in type 2 tympanoplasty*. Eur Arch Otorhinolaryngol.* 2013;270(5):1593-6.
20. Somers T, Van Rompaey V, Claes G, Salembier L, van Dinther J, Andrzej Z, et al. Ossicular reconstruction: hydroxyapatite bone cement versus incus remodelling: how to manage incudostapedial discontinuity*. Eur Arch Otorhinolaryngol*. 2012;269(4):1095-101.
21. Demir UL, Karaca S, Ozmen OA, Kasapoglu F, Coskun HH, Basut O. Is it the middle ear disease or the reconstruction material that determines the functional outcome in ossicular chain reconstruction? *Otol Neurotol.* 2012;33(4):580-5.
22. Dere H, Ozdogan F, Ozcan KM, Selcuk A, Ozcan I, Gokturk G. Comparison of glass ionomer cement and incus interposition in reconstruction of incus long process defects. *Eur Arch Otorhinolaryngol.* 2011;268(11):1565-8.
23. Celik H, Aslan Felek S, Islam A, Demirci M, Samim E, Oztuna D. The impact of fixated glass ionomer cement and springy cortical bone incudostapedial joint reconstruction on hearing results. *Acta Otolaryngol*. 2009;129(12):1368-73.
24. Baglam T, Karatas E, Durucu C, Kilic A, Ozer E, Mumbuc S, et al. Incudostapedial rebridging ossiculoplasty with bone cement*. Otolaryngol Head Neck Surg.* 2009;141(2):243-6.
25. Elsheikh MN, Elsherief H, Elsherief S. Physiologic reestablishment of ossicular continuity during excision of retraction pockets: use of hydroxyapatite bone cement for rebridging the incus*. Arch Otolaryngol Head Neck Surg.* 2006;132(2):196-9.
26. Hafiz G. A more reliable method for incudostapedial rebridging ossiculoplasty: bone cement and wire. *Adv Ther*. 2005;22(1):56-62.
27. Babu S, Seidman MD. Ossicular reconstruction using bone cement. *Otol Neurotol.* 2004;25(2):98-101.
